# Mortality rates in people presenting with a new diabetes-related foot ulcer: a cohort study with implications for management

**DOI:** 10.1007/s00125-024-06262-w

**Published:** 2024-09-27

**Authors:** Naomi Holman, Arthur C. Yelland, Bob Young, Jonathan Valabhji, William Jeffcoate, Fran Game

**Affiliations:** 1 School of Public Health, Imperial College, London, UK; 2 School of Population Health, Royal College of Surgeons in Ireland, Dublin, Ireland; 3 NHS England, Leeds, UK; 4 Diabetes UK, London, UK; 5 Chelsea & Westminster Hospital NHS Foundation Trust, London, UK; 6 Department of Metabolism, Digestion and Reproduction, Faculty of Medicine, Imperial College London, London, UK; 7 Nottingham University Hospitals NHS Trust, Nottingham, UK; 8 University Hospitals of Derby and Burton NHS Foundation Trust, Derby, UK

**Keywords:** Diabetes, Foot ulcer, Mortality

## Abstract

**Aims/hypothesis:**

People with diabetes-related foot ulcers (DFUs) have high mortality rates. This analysis assesses the impact of selected risk factors on short-term mortality using a population registered in the National Diabetes Foot Care Audit (NDFA).

**Methods:**

Mortality rates at 12, 26 and 52 weeks was assessed in people with a new DFU registered by a specialist diabetes footcare service in the NDFA in England and Wales between April 2017 and March 2022. Poisson regression models were created to explore risk factors for mortality.

**Results:**

In 71,000 people registered with a new DFU, mortality rates at 12, 26 and 52 weeks was 4.2%, 8.2% and 14.4%, respectively. At 26 weeks, higher mortality rates was associated with older age (rate ratio 2.15; 95% CI 2.03, 2.28, for age ≥80 years vs age 65–79 years), certain ulcer characteristics (area ≥1 cm^2^ [1.50; 95% CI 1.42, 1.59], deep ulcers [1.26; 95% CI 1.18, 1.35] or hindfoot location [1.53; 95% CI 1.44, 1.62]) and recorded evidence of ischaemia in the lower limb (1.78; 95% CI 1.69, 1.88) and various comorbidities (heart failure [2.13; 95% CI 2.00, 2.26], myocardial infarction [1.45; 95% CI 1.29, 1.63], stroke [1.37; 95% CI 1.22, 1.53], renal replacement therapy [2.34; 95% CI 2.09, 2.61] and chronic kidney disease stage 3 or greater [1.20; 95% CI 1.12, 1.29]). The 26-week mortality rate exceeded 25% for 7.3% of all individuals, rising to 11.5% of those aged 65 years and older, and 22.1% of those aged 80 years and over.

**Conclusions/interpretation:**

Short-term mortality rates in people with a DFU is high. Teams managing people with DFUs should consider modifying the burdensome interventions and care required to heal such ulcers so maximising the quality of residual life, rather than focusing exclusively on healing.

**Graphical Abstract:**

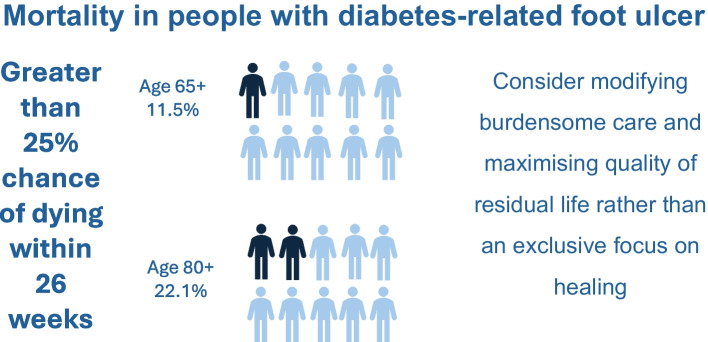


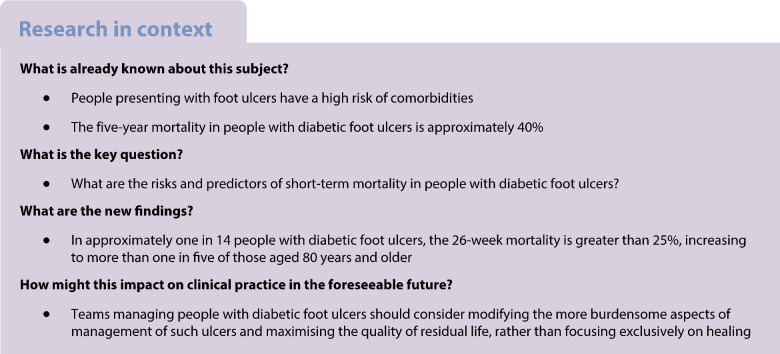


## Introduction

Foot ulceration is a well-recognised complication of diabetes. It is responsible for substantial morbidity, disability and cost [1, 2]. High mortality in people with diabetes-related foot ulcers (DFUs) was first documented 30 years ago [3], and modest reductions were documented up to 2005, possibly associated with improved cardiovascular management [4]. However, more recent reports show that five-year mortality rate for people with diabetes and foot ulceration is still approximately 40%, increasing to 63% in people who have undergone an amputation [5, 6]. A meta-analysis of 11 observational studies included 446,916 people with diabetes, of whom 25,208 had foot ulceration: there were 84,131 deaths overall, and in the pooled random-effects meta-analysis, the relative risk for mortality in people with diabetes who had foot ulceration compared to people with diabetes but without a DFU was 2.45 (95% CI 1.85, 2.85) [7]. The very high mortality rates in those undergoing amputation is greater than that for many common cancers [8]. However, whereas estimation of life expectancy is used to adjust the management plan in the management of cancer, this has not been a routine component of diabetic foot ulcer management [8].

In this study, we have examined the data held by the National Diabetes Foot Care Audit (NDFA) for England and Wales with the aim of clarifying life expectancy for people with a DFU. The NDFA has documented the personal characteristics of people presenting to specialist teams with a DFU, and the characteristics of their ulcers, since 2014 [9]. We aimed to assess the risks and predictors of short-term mortality rates in this population of people presenting with a DFU in order to obtain realistic life expectancy prognoses to inform clinical decision-making. High five-year mortality rate for those presenting with diabetes and an ulcer of the foot has been recognised for a number of years [3, 5, 6], but the potential for high early mortality has not been so widely discussed. The NDFA seeks to establish healing outcomes at 12 weeks in those presenting to a specialist service with a DFU, and, as such, the emphasis has previously been on improving those outcomes on a national basis. This analysis looks at mortality rates over this same period, together with 26-week and 52-week mortality rates, in order that mortality may be included in potential outcomes when formulating treatment plans for patients with a DFU.

## Methods

### Data sources

The core National Diabetes Audit (NDA) has collated data on people with diagnosed diabetes who are registered with a primary care or specialist healthcare practitioner in England and Wales since 2003 [10]. It is based on annual data collection, and individuals are included if they have a valid code for diabetes mellitus (excluding gestational diabetes) in their electronic health record at the time of data extraction. Demographic and clinical data are extracted from general practice electronic clinical systems using the General Practice Extraction Service (a national centralised data collection service for England) and in an annual primary care data submission from Digital Health and Care Wales, and supplemented with data submitted by specialist diabetes services. Each person included in the NDA is identified by a unique National Health Service number that is linked to civil death registrations collated by the Office for National Statistics and Hospital Episode Statistics [11] and the Patient Episode Database for Wales [12], which record all National Health Service inpatient admissions.

The NDFA was established in July 2014 as an additional module of the NDA, and has since continued with collection of data on DFUs. Each specialist diabetic foot service in England is asked to enter data on attendees who present with a new episode of diabetes-related foot ulceration. Data fields include date of first assessment, interval from presentation to any health professional with a new ulcer to the first expert assessment (FEA), ulcer classification at the FEA using the site, ischaemia, neuropathy, bacterial infection, area and depth (SINBAD) classification [13], and whether the person is still alive at 12 weeks after the FEA, and, if so, whether there is still any active foot ulceration.

### Cohorts and observation period

The present study identified a cohort of all people recorded in the NDFA who presented with a DFU between 1 April 2017 and 31 March 2022, and who were followed for 52 weeks after first registration or until death, whichever was sooner. Data from the earlier years of the NDFA were not included due to information governance rules that did not allow linkage to Office for National Statistics mortality records. Where an individual was included in the NDFA more than once, inclusion in the analysis was restricted to the earliest registered episode. The NDFA seeks to include all people with DFU presenting to multi-disciplinary footcare teams in England and Wales.

A secondary cohort of all people included in the 2019/2020 NDA data collection period was identified to facilitate comparison of the demographic and clinical characteristics of people registered with a DFU compared with all people with diagnosed diabetes. The NDA seeks to collect data on all people with diagnosed diabetes in England and Wales. The 2019/2020 data collection included data from 99.2% of general practices in England and 100% of general practices in Wales.

### Demographic and clinical characteristics

The established linkage to the core NDA provided data on age at the FEA, sex, ethnicity, diabetes type, duration of diagnosed diabetes and a cross-border score combining the employment and income domains of the indices of multiple deprivation for England [14] and Wales [15] based on home postcode [16]. eGFR and urine albumin/creatinine ratio measurements included in the NDA were combined with records of renal replacement therapy (RRT) from Hospital Episode Statistics and the Patient Episode Database for Wales, and used to calculate chronic kidney disease (CKD) stage. The severity of the DFU was established using the SINBAD classification for the ulcer at the FEA recorded in the NDFA.

Hospital Episode Statistics and the Patient Episode Database for Wales were used to identify whether individuals had been admitted for myocardial infarction (MI; ICD-10 codes I21–I22; https://icd.who.int/browse10/2019/en), stroke (ICD-10 codes I61, I63–I64 and I679), heart failure (ICD-10 code I50) and RRT (ICD-10 codes N185, Z49, Z992; OPCS codes M01 and X40; https://classbrowser.nhs.uk/ref_books/OPCS-4.9_NCCS-2021.pdf) in the 12 months prior to registration of the DFU at the FEA by the specialist footcare team.

### Outcomes

The outcomes included in this analysis were mortality rates at 12, 26 and 52 weeks after the FEA for the first ulcer registered in the NDFA by the specialist diabetes foot team during the period of study.

### Statistical analysis

The statistical significance of differences between categorical variables was assessed using χ^2^ tests, and the statistical significance of differences between median values was assessed using Kruskal–Wallis tests. Crude mortality rates with 95% CI at 12, 26 and 52 weeks after initial registration at the FEA by the specialist foot team were calculated using the Bryar method [17]. Poisson regression models were created, with mortality at 12, 26 and 52 weeks as the outcome variables. Explanatory variables comprised age, sex, type of diabetes, duration of diagnosed diabetes, ethnicity, socioeconomic deprivation (categorised into quintiles), each of the six elements of the SINBAD classification of the newly registered DFU, whether the individual had been admitted to hospital for MI, stroke, heart failure or RRT in the year prior to DFU registration in the NDFA, and whether the eGFR and urine albumin measurements prior to registration indicated CKD at stage 3 or above. Time as a proportion of the follow-up period was included as the offset variable. The intercepts and coefficients from the Poisson models were used to calculate expected mortality rates for each individual included in the cohort. Hypothetical examples of patients with a DFU are used to illustrate the scale and range of short-term mortality rates in people presenting to specialist services with a DFU. For all statistical tests, a *p* value <0.05 was taken to be statistically significant.

### Information governance

The NDFA and NDA core data are collected and used in line with NHS England’s purposes as required under the statutory duties outlined in the National Health Service Act 2006 and Health and Social Care Act 2012. There is controlled access by appropriately approved individuals to data held in secure data environments entirely within the NHS England infrastructure. Data are processed for specific purposes only, including operational functions, service evaluations and service improvement. The data used to produce this analysis have been disseminated to NHS England under Directions issued under Section 254 of the Health and Social Care Act 2012. Ethics committee approval is not required for these specific purposes. All numbers taken from the NDFA and NDA are rounded to the nearest 5 to protect individuals’ confidentiality.

## Results

A total of 71,000 individuals presenting with a DFU were included in the study. These patients had a median age of 71 years (IQR 60–80); 59,425 (83.7%) had type 2 diabetes and 9520 (13.4%) had type 1 diabetes; the remaining 2050 individuals (2.9%) had other or unknown types of diabetes (Table 1). The median duration of diagnosed diabetes was 15 years (IQR 9–20), with 1945 (2.7%) having a missing date of diagnosis. Ethnicity data were missing for 6790 individuals (9.6%); 58,275 individuals (82.1%) were from White ethnic groups, 3120 (4.4%) were from Asian ethnic groups, 1630 (2.3%) were from Black ethnic groups, and 1190 (1.7%) were from mixed and other ethnic groups. Individuals were not evenly spread across the spectrum of socioeconomic deprivation: 17,250 (24.3%) were living in the most deprived quintile and 9855 (13.9%) in the least deprived quintile; data were missing for 1855 (2.6%).

**Table 1:** Characteristics of all people in the 2019/2020 NDA, the whole cohort who presented with a DFU, and individuals who died within 12, 26 and 52 weeks of first NDFA registration of a DFU

Characteristic	All people in 2019/2020 NDA		Whole cohort with DFU		Died within 12 weeks of registration		Died within 26 weeks of registration		Died within 52 weeks of registration with foot ulcer	
	*n*	%	*n*	%	*n*	%	*n*	%	*n*	%
	3,675,580		71,000		2985		5850		10,210	
Sex										
Male	2,045,930	55.7	48,095	67.7	1835	61.5	3675	62.8	6540	64.1
Female	1,624,055	44.2	21,250	29.9	990	33.2	1900	32.5	3285	32.2
Unknown	5590	0.2	1655	2.3	155	5.2	280	4.8	385	3.8
Age, years										
<40	227,485	6.2	1815	2.6	5	0.2	15	0.3	25	0.2
40–64	1,448,245	39.4	23,645	33.3	330	11.1	660	11.3	1270	12.4
65–79	1,353,615	36.8	26,855	37.8	945	31.7	1995	34.1	3600	35.3
≥80	646,215	17.6	18,640	26.3	1690	56.6	3165	54.1	5290	51.8
Missing	15	0.0	45	0.1	10	0.3	15	0.3	25	0.2
Type of diabetes										
Type 1	298,385	8.1	9520	13.4	225	7.5	475	8.1	890	8.7
Type 2	3,297,880	89.7	59,425	83.7	2585	86.6	5070	86.7	8885	87.0
Other/unknown	79,310	2.2	2050	2.9	175	5.9	305	5.2	440	4.3
Duration of diagnosis, years										
<10	1,932,810	52.6	19,180	27.0	685	22.9	1290	22.1	2250	22.0
10–19	1,284,790	35.0	30,495	43.0	1200	40.2	2415	41.3	4250	41.6
20–29	331,095	9.0	13,650	19.2	655	21.9	1340	22.9	2380	23.3
≥30	108,865	3.0	5725	8.1	275	9.2	505	8.6	900	8.8
Missing	18,020	0.5	1945	2.7	175	5.9	305	5.2	430	4.2
Ethnicity										
White	2,436,010	66.3	58,275	82.1	2300	77.1	4555	77.9	8100	79.3
Asian	455,505	12.4	3120	4.4	105	3.5	205	3.5	360	3.5
Black	165,075	4.5	1630	2.3	55	1.8	110	1.9	210	2.1
Mixed and other ethnicity	110,315	3.0	1190	1.7	50	1.7	85	1.5	140	1.4
Missing	508,670	13.8	6790	9.6	475	15.9	895	15.3	1400	13.7
Socioeconomic deprivation										
Most deprived	875,020	23.8	17,250	24.3	605	20.3	1255	21.5	2265	22.2
2nd most deprived	816,675	22.2	15,175	21.4	610	20.4	1180	20.2	2110	20.7
3rd most deprived	747,560	20.3	14,065	19.8	595	19.9	1135	19.4	2005	19.6
2nd least deprived	659,900	18.0	12,795	18.0	550	18.4	1095	18.7	1885	18.5
Least deprived	536,275	14.6	9855	13.9	455	15.2	890	15.2	1520	14.9
Missing	40,145	1.1	1855	2.6	170	5.7	295	5.0	420	4.1
SINBAD classification										
Area	–	–	34,500	48.6	1865	62.5	3470	59.3	5710	55.9
Bacterial infection	–	–	28,125	39.6	1155	38.7	2230	38.1	3755	36.8
Depth	–	–	11,995	16.9	645	21.6	1260	21.5	2045	20.0
Hindfoot	–	–	13,680	19.3	1045	35.0	1890	32.3	3040	29.8
Ischaemia	–	–	24,795	34.9	1795	60.1	3475	59.4	5785	56.7
Neuropathy	–	–	53,440	75.3	2175	72.9	4310	73.7	7625	74.7
Score ≥3			30,605	43.1	1,785	59.8	3,405	58.2	5,605	54.9
Comorbidities										
Hospital admission for MI	51,465	1.4	1450	2.0	180	6.0	320	5.5	460	4.5
Hospital admission for heart failure	242,580	6.6	8250	11.6	995	33.3	1775	30.3	2820	27.6
Hospital admission for stroke	61,140	1.7	1775	2.5	180	6.0	325	5.6	545	5.3
Hospital admission for RRT	–	–	2385	3.4	245	8.2	455	7.8	760	7.4
CKD stage 0, 1 or 2	–	–	23,235	32.7	985	33.0	1915	32.7	3280	32.1
CKD stage 3 or greater (not on RRT)	–	–	17,650	24.9	1130	37.9	2190	37.4	3890	38.1
Missing CKD data	–	–	30,115	42.4	865	29.0	1745	29.8	3040	29.8

Across the whole cohort, the median SINBAD score was 2 (IQR 1–3), with 30,605 (43.1%) having a score of 3 or more. Within the SINBAD sub-categories, 34,500 (48.6%) had an ulcer greater than 1 cm^2^ in area, 28,125 (39.6%) had evidence of a bacterial infection, 11,995 (16.9%) had a deep ulcer (to muscle/bone), 13,680 (19.3%) had an ulcer on the hindfoot, 24,795 (34.9%) had evidence of peripheral arterial disease (ischaemia), and 53,440 (75.3%) had recorded evidence of neuropathy.

Of all the individuals included in the analysis, 1450 (2.0%) had one or more hospital admissions for MI in the year preceding first registration of the index DFU by the specialist footcare team. The corresponding numbers for heart failure and stroke were 8250 (11.6%) and 1775 (2.5%), respectively. A total of 2385 individuals (3.4%) had received RRT in the year preceding first registration, and 17,650 (24.9%) had evidence of CKD at stage 3 or greater but had not received RRT.

When compared to all people with diabetes included in the 2019/2020 NDA, people in the cohort presenting with a DFU were more likely to be male (67.7% vs 55.7%, *p*<0.0001), older (median age 71 years vs 65 years, *p*<0.0001), and to have a longer duration of diagnosed diabetes (median 15 years vs 8 years, *p*<0.0001). Those with a DFU were also more likely to be of White ethnicity (82.1% vs 66.3%, *p*<0.0001). Although there were statistically significant differences in the distribution across deprivation quintiles in the people with a DFU compared with all people in the 2019/2020 NDA (*p*<0.0001), the differences were not substantial (24.3% of people with a DFU live in the most deprived quintile of areas compared with 23.8% of all people in the 2019/2020 NDA).

Within the cohort of 71,000 people registered with a DFU, 2985 died within 12 weeks (4.2%; 95% CI 4.1, 4.4), 5850 died within 26 weeks (8.2%; 95% CI 8.0, 8.4), and 10,210 died within 52 weeks (14.4%; 95% CI 14.1, 14.6). In those aged 80 years and older at the FEA, the mortality rates at 12, 26 and 52 weeks were 9.1% (95% CI 8.7, 9.5), 17.0% (95% CI 16.5, 17.5) and 28.4% (95% CI 27.7, 29.0), respectively.

Across the whole cohort presenting with DFUs, the expected 12-week and 26-week mortality rate exceeded 25% for 1185 individuals (1.7%) and 5165 individuals (7.3%), respectively. In those aged 65 years and older at the FEA, 2.5% had an expected mortaltiy rate of 25% or greater at 12 weeks and 11.5% had an expected mortality rate of 25% or greater at 26 weeks. In those aged 80 years and older at the FEA, the proportions with an expected mortality rate of 25% or greater were 5.5% at 12 weeks and 22.1% at 26 weeks.

**Fig. 1:**
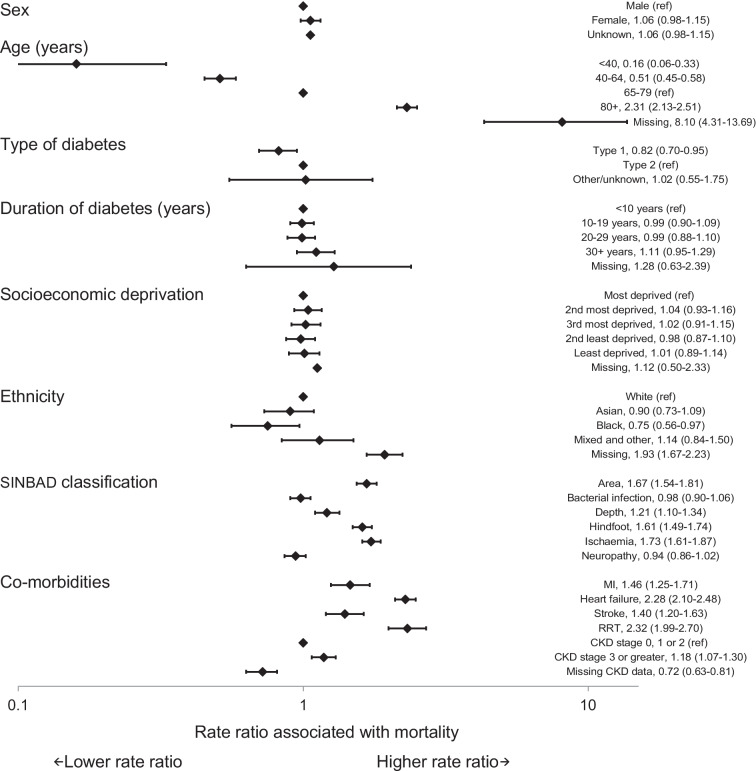
Rate ratios (95% CI) for mortality within 12 weeks of first registration of a DFU with a specialist footcare team

**Fig. 2:**
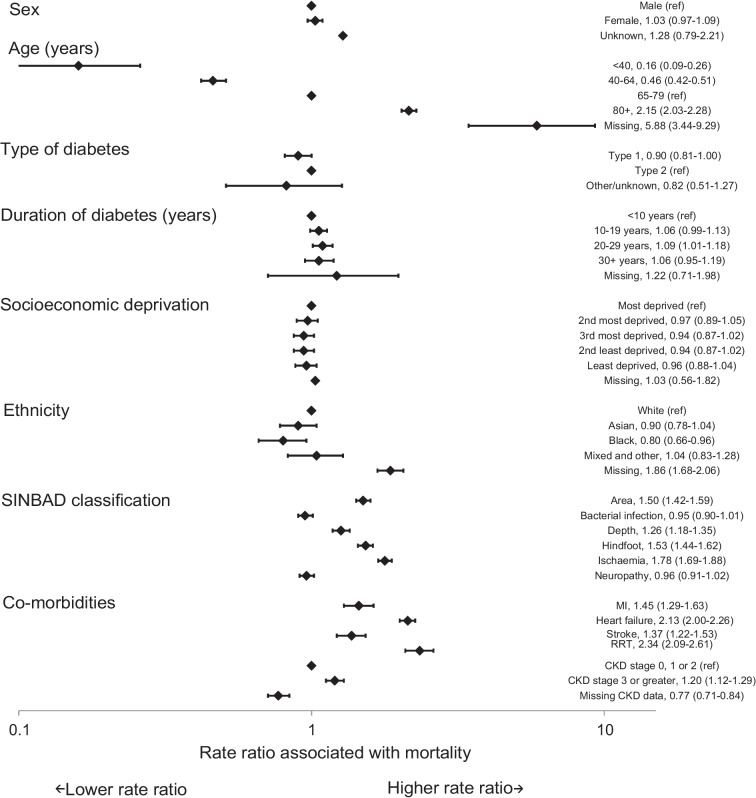
Rate ratios (95% CI) for mortality within 26 weeks of first registration of a DFU with a specialist footcare team

Factors associated with mortality were similar at 12 and 26 weeks (Figs 1 and 2). Older age was the variable most closely associated with higher mortality rates. At 26 weeks, individuals aged 80 years and older had a mortality rate ratio of 2.15 (95% CI 2.03, 2.28) compared with those aged 65–79 years. There were no statistically significant differences in mortality rates by sex, socioeconomic deprivation or duration of diagnosed diabetes at either 12 or 26 weeks. People with type 1 diabetes had lower mortality rates (rate ratio 0.90; 95% CI 0.81, 1.00) than those with type 2 diabetes. People from Black ethnic groups had lower mortality rates than those from White ethnic groups (rate ratio 0.80; 95% CI 0.66, 0.96). Ulcers with an area of 1 cm^2^ or greater (rate ratio 1.50; 95% CI 1.42, 1.59), deep ulcers (rate ratio 1.26; 95% CI 1.18, 1.35), ulcers on the hindfoot (rate ratio 1.53; 95% CI 1.44, 1.62) and evidence of peripheral arterial disease (ischaemia) (rate ratio 1.78; 95% CI 1.69, 1.88) were all associated with a higher mortality rate at 26 weeks. Recorded evidence of bacterial infection and neuropathy were not statistically significantly associated with mortality rates.

**Fig. 3:**
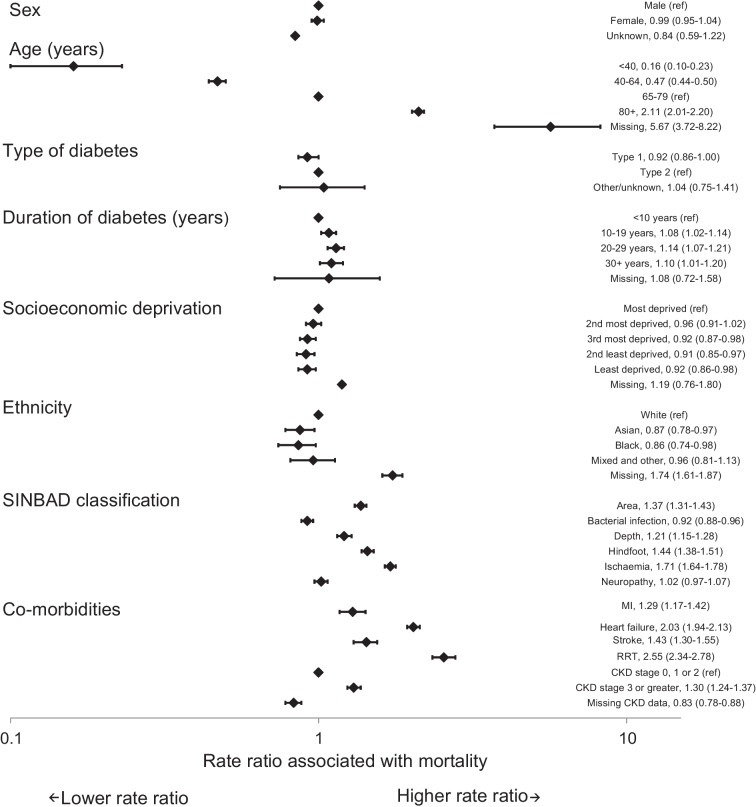
Rate ratios (95% CI) for mortality within 52 weeks of first registration of a DFU with a specialist footcare team

At 52 weeks, older age was also associated with higher mortality rates (rate ratio 2.11; 95% CI 2.01, 2.20 for those aged 80 years and older compared with those aged 65 to 79 years old), but, as at 12 and 26 weeks, there was no statistically significant association with sex (Fig. 3). Type 1 diabetes was associated with a lower mortality rate than type 2 diabetes (rate ratio 0.92; 95% CI 0.86, 1.00); the association between longer duration of diagnosed diabetes and higher socioeconomic deprivation with higher mortality rates reached statistical significance (*p*<0.05 and *p*<0.05, respectively); people from Black and Asian ethnic groups had a lower 52-week mortality rate than White ethnic groups (rate ratio 0.86; 95% CI 0.74, 0.98 and rate ratio 0.87; 95% CI 0.78, 0.97, respectively) and the associations between mortality and ulcer characteristics and cardio-renal comorbidities were similar to those at 12 and 26 weeks.

As an illustration of the divergent outcomes predicted by the model, Table 2 shows the expected mortality rates for four hypothetical people. These hypothetical people are presented as examples but are based on people with similar characteristics seen by the authors.

**Table 2:** Estimated mortality rates for hypothetical people registered with a DFU

Characteristic	Patient 1	Patient 2	Patient 3	Patient 4
Demographic characteristics				
Age group, years	40–59	65–79	≥80	≥80
Sex	Male	Male	Female	Male
Type of diabetes	Type 2	Type 2	Type 2	Type 2
Ethnicity	Asian	White	White	White
Quintile of socioeconomic deprivation	2nd most deprived	3rd most deprived	Most deprived	2nd most deprived
Duration of diagnosed diabetes, years	5–9	20–29	10–19	10–19
Ulcer characteristics				
Ulcer area greater than 1 cm^2^	Yes	Yes	No	Yes
Bacterial infection	Yes	No	Yes	Yes
Ulcer reaching to the muscle, tendon or deeper	Yes	Yes	No	Yes
Ulcer on hindfoot	Yes	No	Yes	Yes
Evidence of ischaemia	Yes	Yes	Yes	Yes
Evidence of neuropathy	Yes	Yes	Yes	Yes
Comorbidities				
Hospital admission for heart failure in past year	No	Yes	No	No
Hospital admission for MI in past year	No	No	Yes	No
Hospital admission for stroke in past year	No	Yes	Yes	No
RRT in past year	No	No	No	Yes
CKD stage 3 or greater	Yes	Yes	Yes	No
Mortality, %				
At 12 weeks	3.3	17.6	16.9	29.8
At 26 weeks	7.0	34.9	37.5	65.2

Patient 1: A man aged 40–59 years from an Asian ethnic group and in the second most deprived socioeconomic quintile who has been diagnosed with type 2 diabetes for 5–9 years and who presents with a foot ulcer with the highest SINBAD score, with no hospitalisation for MI, heart failure, stroke or RRT in the previous year but CKD at stage 3 or greater, has an estimated 12-week mortality rate of 3.3% and a 26-week mortality rate of 7.0%.

Patient 2: A White man aged 65–79 years in the third most deprived socioeconomic quintile who has been diagnosed with type 2 diabetes for 20–29 years who presents with a non-infected forefoot foot ulcer that is over 1 cm^2^ in area and extends to the muscle or tendon, with evidence of ischaemia and neuropathy, a history of hospitalisation for heart failure and stroke in the previous year and CKD 3 or higher, is estimated to have a 12-week mortality rate of 17.6% and a 26-week mortality rate of 34.9%.

Patient 3: A woman aged 80 years or older from a White ethnic group living in one of the most socioeconomically deprived areas, who has been diagnosed with type 2 diabetes for between 10 and 19 years, who has a foot ulcer smaller than 1 cm^2^ on the hindfoot that does not extend to the muscle and tendon, with evidence of ischaemia and neuropathy, with hospital admission for MI and stroke in the previous year and CKD at stage 3 or greater, is estimated to have a 12-week mortality rate of 16.9% and a 26-week mortality rate of 37.5%.

Patient 4: A White man aged 80 years or older in the second most deprived socioeconomic quintile with type 2 diabetes of 10–19 years duration, the most severe foot ulcer (maximum SINBAD score), no hospitalisation for cardiovascular disease but receiving RRT in the previous year, has a 12-week mortality rate of 29.8% and a 26-week mortality rate of 65.2%.

## Discussion

In this analysis of 71,000 people presenting with a new DFU, the 26-week mortality rate was 8.2% overall, but this increased to 17.0% in those aged 80 years and older. The results show that certain characteristics (older age, ulcer severity, cardiovascular and renal comorbidities) that have previously been shown to be associated with risk of death at 5 years [3] apply similarly to short-term mortality rate (at 12, 26 and 52 weeks). For people with multiple high-risk characteristics, life expectancy is very short. Over a quarter of those presenting with a DFU are aged 80 years and older, which is close to current life expectancy for England and Wales [18]. In this age group, 22.1% of people had a 25% or greater chance of dying within 26 weeks.

By comparison with the wider population of people with diagnosed diabetes, people presenting to specialist services with a DFU were older, more likely to be male and from White ethnic groups. These characteristics are generally associated with higher mortality rates in people with diabetes [19]. However, the risk factors for short-term mortality rates in those presenting with a DFU differ from those found amongst the wider group of people with diagnosed diabetes. Thus, in those presenting with a DFU, there was no clear association between 12- or 26-week mortality rates and either the duration of diagnosed diabetes or socioeconomic deprivation, despite their clear associations with mortality rates in the wider population with diabetes [18]. In people with a DFU, the rate ratios associated with type of diabetes (statistically significant) and sex (not statistically significant) are the inverse of those found when examining mortality rates in all people with diabetes [18].

The consistency with which high mortality rates and the risk factors for mortality in people with a DFU have been identified in diverse studies over more than a decade supports their validity. The main risk factors relate to age, comorbidities and ulcer severity rather than type and duration of diabetes and social deprivation (see Figs 1, 2 and 3). These findings imply that, although demographic, social and environmental factors may play a role in the early development of a DFU, life expectancy at presentation of a DFU to specialist services is more a product of underlying disease processes. The question that subsequently arises is whether this knowledge about life expectancy and mortality risk factors should influence the management plans for individuals? First, the pattern of mortality risk factors implies an obligation to broaden the management plan to include optimisation of cardiovascular risk reduction and disease management with statins, use of glucose-lowering agents that benefit and do not increase cardiovascular disease risk, and management of blood pressure, arrhythmias and heart failure. Second, a dominant issue in people with a DFU is often renal disease, implying a need for coordination with renal specialist services. Third, the threat in another group may arise from factors that contribute to the risk of developing a DFU itself (especially peripheral vascular disease). These factors imply that the management plan for people with a DFU needs to be broader than focusing solely on foot ulcer healing. Specialist foot ulcer services have a potentially pivotal role in making sure that the complex care requirements of these people are accommodated. Furthermore, for those with the shortest life expectancies, consideration should perhaps be given to directing care away from the more burdensome aspects of management towards palliation and maximising quality of residual life rather than focusing exclusively on healing.

Palliative care helps people, and their families, live as full and as comfortable a life as possible while facing a life-limiting or terminal illness. Palliative care may be implemented at any stage of incurable illness, and has previously been advocated for people with diabetic foot disease [20]. Given that the 26-week mortality rate is greater than 25% for about 1 in 14 of all those presenting with a DFU (rising to more than one in five of those aged 80 years and older), our analysis suggests that a palliative approach to foot ulcer management plan should be considered more frequently, especially in those patients who have similar characteristics to the scenarios presented in Table 2, where expected mortality rate may exceed 60% over 12 weeks. As the use of patient decision aids to support shared decision making for patients and their carers in making health treatment decisions becomes more widespread, it is important that we have robust data to inform these decisions, including an understanding of life expectancy. Discussions with patients should therefore include enhanced understanding and acceptance of the prognosis in order to help frame the scope, location and limitations of interventions and to guide choice of the most appropriate support systems.

The strength of this study lies in the large size of the cohort of people with a DFU, which allows identification of risk factors for mortality over a short time period and highlights how mortality rates evolves in the year following initial presentation to specialist services. However, a limitation is that we do not have accurate data on the total number of people with a DFU. Therefore, we cannot be certain of the proportion of people with a DFU included in the NDFA. Accordingly, our observations are restricted to people attending participating specialist services.

We conclude that short-term mortality rates in older people with a DFU and cardiovascular or renal comorbidities is very high. Mortality is also clearly associated with ulcer position, area and depth. Better understanding of the significance of these factors for life expectancy can inform the choice of a holistic approach to the delivery of overall care in this increasingly elderly group.

## Data Availability

Data from the National Diabetes Audit and the National Diabetes Foot Care Audit may be obtained via the Data Request Service at NHS England (see https://digital.nhs.uk/services/data-access-request-service-dars).

## Abbreviations

CKD: Chronic kidney disease
DFU: Diabetes-related foot ulcer
FEA: First expert assessment
MI: Myocardial infarction
NDA: National Diabetes Audit
NDFA: National Diabetes Foot Care Audit
RRT: Renal replacement therapy
SINBAD: Site, Infection, Neuropathy, Bacterial infection, Area, Depth

## Authors’ relationships and activities

JV is grateful for support from the North West London National Institute for Health Research (NIHR) Applied Research Collaboration, the Imperial NIHR Biomedical Research Centre, and CW+, the official charity of Chelsea and Westminster Hospital NHS Foundation Trust. JV was National Clinical Director for Diabetes and Obesity at NHS England from April 2013 to September 2023, is currently the National Clinical Lead for Multiple Long-Term Conditions (MLTC) at NHS England, and is North West London NIHR Applied Research Collaboration Theme Lead for MLTC. FG is the current Clinical Lead for the NDFA, and WJ is a former Clinical Lead for the NDFA. BY is the Clinical Lead for the NDA. NH, BY and JV are members of the NDA Research Advisory Group. The other author declares that there are no relationships or activities that might bias, or be perceived to bias, their work.
